# 

**Published:** 1881-11

**Authors:** 


### CHICAGO MEDICAL Journal and Examiner

**EDITORS:**

**N. S. DAVIS, M.D., LL.D. JAS. NEVINS HYDE, A.M., M.D.**

**DANIEL R. BROWER, A.M. M.D.**

S.G.O. INDEXED

CHICAGO MEDICAL PRESS ASSOCIATION.

AUG. 2D

1875.

CHICAGO:

*PUBLISHED BY THE CHICAGO MEDICAL PRESS ASSOCIATION,*

188 CLARK STREET.

☞ **Read the Notice of this Journal among Advertisements.**

